# Mental Pain Responses of Ultramarathon Runners: A Scoping Review

**DOI:** 10.3390/sports14020072

**Published:** 2026-02-06

**Authors:** Marie Delalay, Sabina Hotz Boendermaker

**Affiliations:** Institute of Physiotherapy, School of Health Professions, Zurich University of Applied Sciences ZHAW, 8400 Winterthur, Switzerland; sabina.hotzboendermaker@zhaw.ch

**Keywords:** pain, ultramarathon, mental pain responses

## Abstract

Ultramarathon runners experience pain during the race. Their mental responses to pain influence race performance. This scoping review synthesises the existing literature on the mental pain responses of ultramarathon runners. The framework of the Preferred Reporting Items for Systematic Reviews and Meta-Analyses guidelines for scoping reviews (PRISMA-Scr) was followed. We screened four online databases, obtaining 121 non-duplicate publications. We filtered these publications to eventually include seventeen research articles. Results were structured according to four overarching categories: thought processes, psychological traits, pain expectations, and cultural discourses. Ultramarathon runners have both associative and dissociative thoughts in response to pain. They are less harm avoidant and less anxious about pain than the general population. They expect and accept pain. Their mental pain responses are modulated by ultra-running discourses. While mental pain responses of ultramarathon runners have been moderately described in the literature, their effects on race performance remain largely unknown. This represents an exciting opportunity for future research.

## 1. Introduction

### 1.1. Definitions and Objectives

Ultramarathon races are running events of a distance longer than the traditional 42.195 km marathon [1]. Having gained popularity in many parts of the globe in the last two decades, they encompass a large variety of conditions: freezing cold at high altitudes to burning hot in deserts; solo or relay races; on mountain trails, sandy ways or loop running tracks; as single-stage or multi-stage events; and covering 50 to more than 300 km per stage [1,2,3].

To enhance both personal race experience and race performance, ultramarathon runners must manage their pain effectively [1,4,5,6]. Their pain and pain sources include pre-existing injuries, injuries during the race, blisters or “hot spots” on the feet, muscle cramping, and muscle pain [7]. Pain is more prevalent and/or more severe in non-finishers than finishers [4].

Many biopsychosocial factors modulate pain in ultramarathon runners, such as fitness level, race experience, level of preparation to race, motivations for race participation, and pain responses [2,8,9,10,11,12,13]. Important motivations are achieving personal goals, pushing one’s limits for self-discovery or self-esteem, and enjoying the present moment [2]. Pain responses are physiological (e.g., changes in peripheral and central pain mechanisms), behavioural (e.g., pace reduction and medication use), and mental (e.g., associative thoughts and pain acceptance) [4,6,8,14,15]. When pain responses are effective, they typically lead to a reduction in pain and/or damage.

To date, no review on the mental pain responses of ultramarathon athletes exists. Therefore, this scoping review aims to identify and synthesise the existing literature on the nature of mental pain responses of ultramarathon runners and their effects on performance. The gained insights will increase the body of knowledge in sport science, pain science, and psychology. Also, they will contribute to more effective mental pain responses of ultramarathon athletes—which enhance both race performance and personal race experience [1,5,6].

### 1.2. Theoretical Background

Mental pain responses can be split into thought processes, psychological traits, pain expectations, and cultural discourses [2,11,14,16].

Owing to the intricate connection between emotional and cognitive processes [17], we do not distinguish between them. Rather, we put mood, thoughts, feelings, emotions, and mental attitude together, referring to this aggregate as thought processes. We distinguish between associative and dissociative thoughts, which we also refer to as association and dissociation techniques [17]. When runners have associative thoughts, they think about the run. For example, they focus on physical feelings and sensations, rely on interoception for pacing, and keep telling themselves to relax. By contrast, when runners have dissociative thoughts, they think about topics not related to the run. For example, they recall childhood memories, visualise past sport achievements, and concentrate on music. Runners are prone to having dissociative thoughts when they need a distraction from pain and discomfort.

Mental pain responses are influenced by psychological traits. Psychological traits are studied via questionnaires and inventories. For example, the Temperament and Character Inventory measures individual differences in temperament and character. It includes seven dimensions, such as Novelty Seeking, which is the tendency to seek novel stimuli; Harm Avoidance, which is the tendency to avoid or cease behaviours in response to aversive stimuli; Reward Dependence, which is the tendency to respond intensively to sentimental or social reward; Cooperativeness, which accounts for individual differences in acceptance of and identification with other people; and Self-Transcendence, which refers to identification with something beyond the self that gives existential meaning [18]. A questionnaire for the study of psychological traits is the Pain Anxiety Symptoms Scale-20. It measures anxiety and fear responses to pain across cognitive, behavioural, and physiological domains. It consists of 20 statements and four subscales—cognitive anxiety, fear of pain, escape and avoidance behaviour, and physiological anxiety [12]. Psychological traits also relevant for the mental pain responses of ultramarathon runners are sport-specific trait boredom and mental toughness. Sport-specific trait boredom relates to boredom associated with sports and exercise [19]. Mental toughness is a trait characterised by the ability to ignore, control, accept, overcome, endure, embrace, and celebrate pain; to listen to the body; and to distinguish between “regular pain” and “injury pain” [20].

Mental pain responses are also modulated by pain expectations. The object of pain expectations is the expected intensity, duration, and quality of future pain. In the general population, pain expectations affect decision making [21] and pain intensity [22]. In athletic populations, they influence the pain experience [23].

Finally, mental pain responses are influenced by ultra-running discourses. Discourses describe sets of ideas that “can act to constrain and subject people to certain ends, identities, and modes of behaviour” [14]. Grounded in normative principles and activities, cultural discourses evolve in communities and shape these communities, which are concrete (e.g., a local running group) or abstract (e.g., the running community) [24]. Cultural discourses gear individuals towards the norm and provide them with a meaningful interpretation of experiences. Via cultural discourses, ultramarathon runners obtain a sense of belonging to communities, create their identity, and in a feedback loop reinforce discourses.

## 2. Materials and Methods

The methods drew upon the framework of the Preferred Reporting Items for Systematic Reviews and Meta-Analyses guidelines for scoping reviews (PRISMA-Scr) [25]. The completed PRISMA-ScR Checklist is provided in the Supplementary File S1. In consultation with research librarians, articles in four online databases—PubMed, Science Direct, Scopus, and Web of Science—were screened using queries with the terms “ultramarathon” and “pain” (Table 1). Dates ranged from 1 January 1900 to 29 June 2025, and the language was restricted to English. In addition, backward and forward citation search was carried out, thereby constituting the fifth data source. At the end of this stage, the number of publications was 216. After duplicate removal, it was 121.

**Figure 1:**
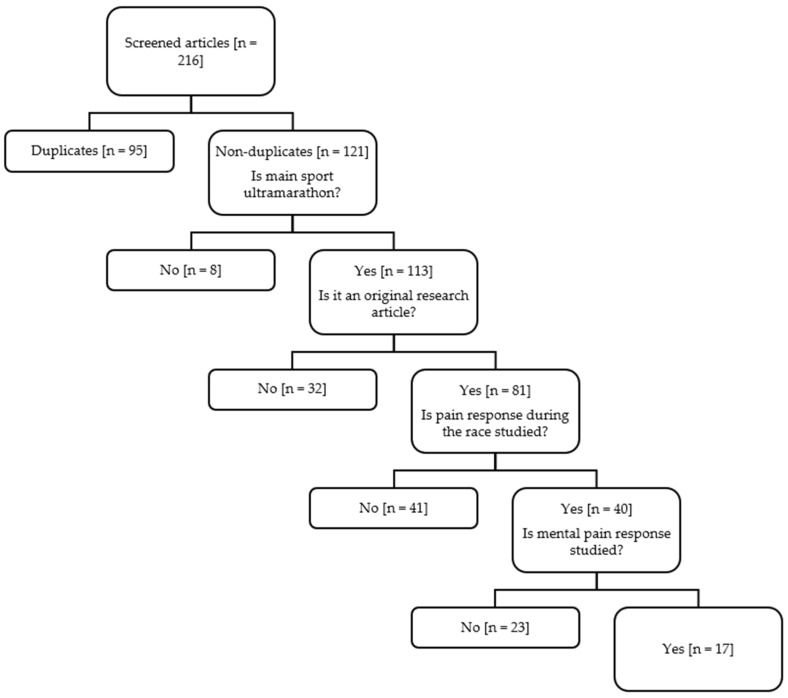
Flow diagram of selected studies.

We selected 17 original research articles that pertained to mental pain responses during ultramarathon races. To do so, we filtered the 121 non-duplicate publications in a pre-defined sequence (Figure 1). First, we retained 113 articles as they primarily focussed on ultra-running, discarding, for instance, articles on triathletes. Second, we selected 81 studies as they were peer-reviewed original research articles, thus removing reviews, conference abstracts, and book chapters. Third, we kept 40 studies as they covered pain responses during the race, excluding, for example, studies solely on pain responses during training. Finally, after discarding studies that looked only into physiological or behavioural pain responses, we obtained 17 studies on mental pain responses during ultramarathon races.

Relevant information from the included studies was charted in Excel. We recorded the study’s objectives, research design, findings about mental pain responses, and count of study participants. If the study pertained to a specific race, we also collated the race name, location, distance, ascent, duration, and type of terrain.

During data charting, we also assigned findings to one of four overarching categories—thought processes, psychological traits, pain expectations, and cultural discourses. These four overarching categories emerged from the authors’ discussions about distinctions and commonalities of the included studies. These categories agree with the existing literature, according to which mental pain responses manifest themselves as the combination of emotional and cognitive processes, and are modulated by psychological traits, pain expectations, and cultural discourses [2,11,14,16].

## 3. Results

### 3.1. Study Characteristics

The charted data is presented in Table A1. Nine studies are observational, and eight are phenomenological. The distribution of the number of study participants is right skewed (median = 22, mean = 80, standard deviation = 122). Ten studies are not race-specific. The seven remaining studies take place in mountains, deserts, and loop running tracks in all five continents; with distances from 65 km to 330 km and elevation differences up to 24,000 m; and in solo and in relay teams. Four studies focus primarily on pain, while thirteen look into pain only in relation to their primary focus—such as injuries and personal race experience.

### 3.2. Thought Processes

Ten studies report on thought processes in response to pain [4,16,20,24,26,27,28,29,30,31]. Based on our synthesis of the studies, (1) ultramarathon athletes have pain-related and pain-unrelated associative thoughts as well as dissociative thoughts in response to pain; (2) they resort to pain denial; and (3) association and dissociation techniques influence performance in various, sometimes contradictory ways.

First, ultramarathon athletes have pain-related associative thoughts in response to pain. In Alschuler, Kratz [16] and Alschuler, Krabak [4], participants spend 31% (±21%) of their race time having pain-related associative thoughts. In Alschuler, Kratz [16], pain-related thoughts are more often positive (e.g., participants see pain as a challenge) than negative (e.g., they feel that pain is unbearable). Alschuler, Kratz [16] calculate a positive correlation between the time spent with pain-related thoughts and the index for experiential awareness, which quantifies the extent to which runners are optimistic, want to continue, feel a sense of automaticity, and observe mindfully. Also, pain-related associative thoughts can pertain to pain interpretation. In Antonini, Rochat [27], ultramarathon runners seek explanations for their pain in various domains, such as their physiological and mental states (e.g., injuries and own resources), the physical environment, and the weather conditions. Likewise, in Rochat, Gesbert [28], ultramarathon runners search for the causes and origins of their pain. Hall and Rhodes [24] suggest that ultramarathon runners iteratively look into and respond to pain until this becomes superfluous, for example, when pain is relieved or harmless, it is physiologically explained, or it has a meaning in light of the runner’s internalised discourses.

Ultramarathon runners have pain-unrelated associative thoughts in response to pain. Study participants in Simpson, Post [26] and Holt, Lee [30] respond to pain by maintaining an optimistic inner dialogue, setting manageable goals, visualising positive events, paying attention to their running style, engaging in mental and physical battles, monitoring nutrition, and avoiding dissociative thoughts.

Ultramarathon runners rely on dissociative thoughts as well, mainly to distract themselves from difficult or painful moments in the race. For example, runners in Antonini, Rochat [27] and Holt, Lee [30] attempt to distract themselves from pain by listening to music and receiving support from family, friends, fellow racers, and race staff. Notably, Kirkby [31] estimates that only 29.4% of the thoughts of an ultramarathon participant are dissociative (the rest being associative) and that pain explains 90% of mood variance.

Second, ultramarathon runners deny pain. Because pain is the symptom most commonly reported (80.1% of the respondents) to be associated with overuse injuries in ultramarathon runners, Wickström, Spreco [29] infer that ignoring pain and neglecting long-term implications are key risk factors for overuse injuries. Also, for some ultramarathon runners in Hall and Rhodes [24], pain elicits a fear of pain, a fear of death, and a feeling of physical betrayal. Runners respond to these emotions by denying pain and alienating their painful body and, as such, relying on a mind-body dualistic approach to regain a sense of agency over a reified pain. Last, in Jaeschke, Sachs [20], ultramarathon runners ignore physical and mental signals such as discomfort, fatigue, and pain.

Third, while both association and dissociation techniques are used to respond to pain, these techniques influence race performance in various, sometimes contradictory, ways. In Holt, Lee [30], ultramarathon finishers have more associative thoughts than non-finishers. Possibly in contradiction, in Alschuler, Kratz [16], a higher index for experiential awareness coincides with more pain interference. Also, more negative pain-related thoughts increase the odds of withdrawal, while neither positive nor negative pain-related associative thoughts are linked to race ranking [4]. Finally, two studies report that more thoughts geared towards pain interpretation are associated with performance deterioration. In Antonini, Rochat [27], pain interpretation belongs to one of several sequences that precede withdrawal. In Hall and Rhodes [24], pain interpretation is part of a personal evaluation of the risk of performance-deteriorating pain and injury.

### 3.3. Psychological Traits

Five studies report on psychological traits of ultramarathon runners in the context of mental pain responses [11,12,19,20,32]. Based on our synthesis of the studies, (1) ultramarathon runners are less harm avoidant and less anxious about pain than the general population; (2) pain perception could be associated with psychological traits; (3) sport-specific trait boredom and performance are associated; and (4) ultramarathon runners have high levels of mental toughness, which are linked to the ability to respond effectively to pain.

First, ultramarathon runners are less harm avoidant and less anxious about pain than the general population. In Freund, Weber [11], overall scores of the Temperament and Character Inventory that are related to the dimensions Harm Avoidance, Reward Dependence, and Cooperativeness are significantly lower for ultramarathon runners than for controls, while the overall score related to the dimension Self-Transcendence is higher. Also, in Roebuck, Urquhart [12], ultramarathon runners have lower scores on all four Pain Anxiety Symptoms Scale-20 subscales than the control group.

Second, pain-tolerance-level stimuli are correlated with scores related to psychological traits, suggesting that pain perception is associated with psychological traits. In Freund, Weber [11], lower pain ratings at the end of the cold pressor test are associated with scores related to psychological traits like more Novelty Seeking, less Reward Dependence, and less Cooperativeness. In Roebuck, Urquhart [12], pain-tolerance-level stimuli increase with lower scores on the subscale for pain escape and avoidance behaviour. The authors infer that pain perception is associated with psychological traits. They hypothesise that a higher pain tolerance level is partially mediated by psychological traits, while acknowledging that causes of associations between pain ratings, pain tolerance level, psychological traits, and ultra-running activities remain largely unknown.

Third, sport-specific trait boredom and performance are associated. In a study investigating boredom, pain, willpower, and effort in a 24 h running track race, “very extreme” ultramarathon runners (i.e., solo runners and runners in a relay team of two) display a significantly lower sport-specific trait boredom than less extreme ultramarathon runners (i.e., runners in relay team of four or six) [19].

Fourth, mental toughness in the context of ultra-running is linked to effective mental pain responses, and mental toughness levels are high in ultramarathon runners. Measured with the Sports Mental Toughness Questionnaire, mental toughness levels in Brace, George [32] are higher in ultramarathon runners than in both athletes from other sports and non-athletes. The authors suggest that pain perception is associated with mental toughness, hypothesising that higher levels of mental toughness lead to exercise-induced endogenous hypoalgesia via an increased efficiency of opioid receptors.

### 3.4. Pain Expectations

Four studies report on pain expectations in the context of the mental pain responses of ultramarathon runners [14,16,26,33]. Based on our synthesis of the studies, (1) ultramarathon runners expect and accept pain and (2) increased expectation and acceptance of pain is associated with less negative thoughts during the race.

Ultramarathon runners expect and accept pain. In Simpson, Post [26], ultramarathon runners believe that expecting and accepting a certain level of pain allows them to better cope with it. In Hanold [14], “good pain” is defined as the pain that ultramarathon participants expect and could tackle effectively, for example, by being tough and patient. Illustratively, even though the study participant in Cherrington, Black [33] describes his pain during an ultramarathon to be “more visceral” than that during standard exercise, he accepts it “as a welcome companion” which he must endure. Finally, in Alschuler, Kratz [16], ultramarathon runners with increased pain expectation and acceptance are less likely to have negative thoughts (e.g., distress, fear, and unpleasantness)—they accept pain because they recognise its inevitability for race completion.

### 3.5. Cultural Discourses

Six studies report on pain experience, meaning, and response from the vantage point of communities and cultural discourses of ultra-running [14,24,28,30,33,34]. Based on our synthesis of the studies, (1) ultra-running discourses modulate pain meaning and response, and (2) they might give rise to a community united by pain.

Honouring endurance, self-reliance, strength, and heroism, as well as normalising pain and suffering, ultra-running discourses modulate pain meaning and response [24]. For example, the study participant in Cherrington, Black [33] accepts pain because it gives him the chance to “respond, endure, and learn [his] true self-worth”. In Bill and Antonini [34], experiencing pain and suffering gives ultramarathon athletes a sense of pride and joy, boosts self-confidence, and enables self-discovery. In search of social recognition, endurance runners often instrumentalise their experiences of pain and injuries [24]. For example, they emphasise honoured and normalised qualities when telling about their running experiences [24]. Cultural discourses constrain the body to tolerate discomfort and pain [14].

Ultra-running discourses might give rise to the pain community. Ultramarathon athletes often look for their “tribe”, a community of like-minded people in which they are accepted and feel normal [14,34]. The pain community, i.e., a community united by pain, could bring together triathletes and fell runners as they share physical and mental suffering through self-exploration and a collective quest [28]. In the same way, ultra-running discourses might give rise to the pain community of ultramarathon runners. Possibly owing to a sense of belonging to the pain community, ultramarathon runners encourage each other in difficult moments [30]. In most sports, if competitors support the performance of each other at all, it is realised via competition against each other. Competitors as a source of support might, therefore, be unique to ultra-endurance sports [30].

## 4. Discussion

### 4.1. Future Research Direction

As a summary of the results, (1) ultramarathon runners have both associative and dissociative thoughts in response to pain, (2) they are less harm avoidant and less anxious about pain than the general population, (3) they expect and accept pain, and (4) their mental pain responses are modulated by ultra-running discourses. While providing insight, the results also hint at the need for future research.

Ultramarathon runners use dissociation techniques to respond to pain. Yet, dissociation techniques can be detrimental to function. For example, they can distract ultramarathon runners from interoceptive and emotional experiences at a time when these experiences are essential for self-regulation [13].

Pain denial coincides with overuse injuries. As overuse injuries are extremely common in ultramarathon runners, pain denial as a mental pain response must, therefore, be extremely common as well [5]. This suggests that pain-related associative thoughts could prevent overuse injuries.

In Alschuler, Kratz [16], a higher index for experiential awareness coincides with more pain interference. Assuming that (1) more awareness is associated with more associative thoughts and (2) pain interference causes performance deterioration, this suggests that more associative thoughts lead to performance deterioration. This contradicts the existing literature, according to which more awareness in a sport enhances performance, for example, via emotional regulation [13]. This discrepancy could be explained by differences in pain intensity and duration between ultramarathon runners on one hand and athletes considered in the existing literature on the other hand. In ultra-running activities, the average pain intensity is higher, and pain duration is longer than in many other sports. Sensations and experiences captured in an aware state are mostly positive for athletes from these other sports and, as such, enhance performance. By contrast, sensations and experiences (e.g., pain) can be negative for ultramarathon runners and, as such, deteriorate performance [16].

Future research is needed to understand (1) associations between thought processes (e.g., associative and dissociative thoughts) and race performance and (2) mechanisms underlying these associations [1,26]. To do so, researchers will have to be consistent in how they categorise thought processes and measure performance.

Thought processes, mental pain responses, and psychological traits of ultramarathon runners are associated. Previous research suggests that acute mood effects during ultra-running activities coincide with an increase in fatigue and a decrease in vigour and tension; that the primary motivation of ultramarathon runners is the opportunity to achieve personal goals; and that ultramarathon runners are eager to explore their physical and mental limits [2]. Future research is needed to determine causal relationships between thought processes and psychological traits, as well as the role of psychological traits in mental pain responses.

Ultramarathon runners expect and accept pain. They view pain as a normal experience and worry about actual pain only if it differs from expected pain [2]. Their extraordinary resistance to pain might owe to their mindful acceptance of pain. A pain that is expected and that has less uncertainty is associated with less negative thoughts compared with (1) a pain that is unexpected or (2) an expected pain that has more uncertainty. Pain is better coped with when, in expectation or in fact, pain gives intrinsic rewards (e.g., personal achievement, identification with a group) [13]. Future research is needed to explore causes of associations between thought processes and pain expectations, as well as the role of pain expectations in mental pain responses.

Ultra-running discourses modulate mental pain response. The meaning given to pain and the need to belong to the pain community could explain why ultramarathon runners opt for enduring so much pain for so long. By changing the meaning ascribed to pain, ultramarathon runners can transform negative sensations into positive ones [13]. In addition, the pleasure that ultramarathon athletes find in extreme bodily discomfort and hardship could be acquired as part of a ritualistic process [13]. This process grants access to the ultramarathon community that is united by pain experience. Interestingly, Johnson, Hudson [35] argue that the current metaphorical language about pain—one of the many conveyors of pain discourses—promotes pain persistence; that it is insidious, damage-loaded, and “warmongering”; and that it governs the explanation and meaning that people give to their pain. This view questions whether dominant pain discourses in ultra-running are beneficial at all [13]. Future research is needed to determine risks and benefits of pain discourses in ultra-running, as well as to examine the influence of ultra-running discourses on mental pain responses.

Not only effective mental pain in particular, but also effective pain management strategies in general, enhance personal race experience and race performance. As such, future research on the mental pain responses of ultramarathon runners should be embedded in research on pain management strategies of ultramarathon runners. In addition to mental pain responses, these strategies include behavioural and physiological pain responses, as well as fitness level, race experience, level of preparation to race, and motivations for race participation [2,8,9,10,11,12,13].

### 4.2. Limitations

This scoping review has a few limitations. First, only four studies primarily look into mental pain responses of ultramarathon athletes. The remaining studies treat pain as an adjacent topic to their primary topics, and, as such, their analyses and discussions are not centred on mental pain responses. For instance, no study focuses primarily on the role of pain expectations in mental pain responses. Second, all studies adopt an observational or phenomenological research design, which prevents the establishment of causal mechanisms. Third, definitions of cognitive and emotional processes (i.e., mood, thoughts, feelings, emotions, and mental attitude) vary across studies. For example, associative thoughts explicitly serve to maintain awareness in Holt, Lee [30], while Alschuler, Krabak [4] propose an index of experiential awareness without referring to associative and dissociative thoughts. Therefore, we have synthesised the studies through the lens of our own definitions of cognitive and emotional processes, which may have caused a slight loss of information.

Last, propagated in this scoping review, two limitations of the reviewed studies must be mentioned. First, many of the studies have small sample sizes, with half of the studies relying on a sample of 22 subjects or less. A smaller sample size increases the risk that (1) the sample is not representative of the ultra-running population investigated in the study and (2) existing effects are missed (i.e., Type II statistical errors) [36]. Second, risk of selection bias exists in most studies. It is possible that ultramarathon runners who participate in a scientific study have common characteristics related to mental pain responses which the ultra-running population at large does not.

## 5. Conclusions

This scoping review has included 17 studies on mental pain responses of ultramarathon runners. Findings were split into four overarching categories—thought processes, psychological traits, pain expectations, and cultural discourses. This work shows that (1) mental pain responses of ultramarathon runners have been moderately described in the literature, and (2) all studies are observational or phenomenological, and, as such, cannot establish the causes of associations between mental pain responses and race performance. Yet, these causes of associations are key to determine effective mental pain responses. This represents an exciting opportunity for future experimental research.

## Figures and Tables

**Table 1:** Publication count resulting from screening of online databases, with and without duplicates.

Data Source	Query	Count, as Retrieved	Count, After Duplicate Removal
PubMed	(ultramarathon [Title/Abstract]) AND (pain [Title/Abstract])	52	50
ScienceDirect	Title, abstract, keywords:ultramarathon pain	8	2
Scopus	(TITLE-ABS-KEY (ultramarathon) AND TITLE-ABS-KEY (pain))	50	13
Web of Science	(TS = ultramarathon) AND (TS = pain), in All databases	68	18
Backward and forward citation search	Article title	38	38
Total		216	121

## Data Availability

Data sharing not applicable to this article as no datasets were generated or analysed during the current study.

## Supplementary Materials

The following supporting information can be downloaded at: https://www.mdpi.com/article/10.3390/sports14020072/s1, File S1: PRISMA Checklist for ScR.

## Appendix A

**Table A1:** Charted data from included studies. Rows are ordered by research design, publication year, and first author. Column “Study participants” indicates the number of study participants.

Authors	Objectives	Race Description	Study Participants	Research Design
Weich, Schüler [19]	To investigate the association between sport and boredom in ultramarathons; To determine whether and how boredom predicts a crisis for ultramarathon athletes during the race	24 h race on a 3.5 km running track, Germany; actual best distance = 197 km	107	Cross-sectional study
Alschuler, Krabak [4]	To examine the type of pain coping strategies used during ultramarathon races and their effects on performance	Five stages of the 2016 RacingThePlanet 250 km six-stage ultramarathon races in the Atacama Desert in Chile, Gobi Desert in China and Namibian Desert; 40 km (first four stages) and 80 km (fifth stage)	204	Cross-sectional study
Wickström, Spreco [29]	To investigate perceptions of overuse injury among long-distance runners with different exercise loads	not race-specific	165	Cross-sectional study
Roebuck, Urquhart [12]	To examine pain-related psychological processes in ultramarathon runners; To explore the association between psychological factors and the elevated pain tolerance of these runners	not race-specific	40	Cross-sectional study
Freund, Weber [11]	To compare pain tolerance and personality traits between ultramarathon athletes and a control group	not race-specific	22	Cross-sectional study
Brace, George [32]	To examine how mental toughness and self-efficacy are associated with performance in ultramarathon runners	Hawaiian Ultra Running Team’s Trail 100-mile; 161 km of mountain trails in Hawaii; 7620 m of ascent	56	Case-series study
Alschuler, Kratz [16]	To examine reported pain intensity and pain coping strategies during an ultramarathon; To investigate how stage-to-stage changes in pain coping strategies relate to both the amount of time spent thinking about pain and the amount of pain-related interference	Five stages of the 2016 RacingThePlanet 250 km six-stage ultramarathon races in the Atacama Desert in Chile, Gobi Desert in China and Namibian Desert; 40 km (first four stages) and 80 km (fifth stage)	204	Case-series study
Jaeschke, Sachs [20]	To explore how ultramarathon runners perceive mental toughness	not race-specific	468	Case-series study
Kirkby [31]	To investigate mood changes and cognitive processes of an elite runner during a 48 h race	48 h running race	1	Single-case report
Hall and Rhodes [24]	To understand the socio-cultural discourses that are resisted and reinforced through ultramarathon runners’ experiences of injury; To identify difficult or hidden experiences that are revealed by injury	not race-specific	8	Phenomenological study
Bill and Antonini [34]	To explore the long-term individual journeys of ultra-endurance athletes and how their passion develops over time from the moment of inception to full adoption of ultra-endurance as an identity and a lifestyle	not race-specific	16	Phenomenological study
Cherrington, Black [33]	To explore how ultramarathon participants begin appreciating the immense power of nature, while being humbled by the fragile and unstable foundations of human experience	not race-specific	1	Phenomenological study
Rochat, Gesbert [28]	To identify phenomenological characteristics shared by ultramarathon runners; To explore meaningful activities of runners during the race	Tor des Géants, France and Italy; 330 km of mountain trails; 24,000 m of ascent; Cut-off time of 150 h	10	Phenomenological study
Antonini, Rochat [27]	To report on the experience of race withdrawal by ten ultramarathon runners	Grand Raid de la Réunion; 65 km (short race), 97 km (middle race) and 173 km (long race) of nature trails; 3922 m (short), 5655 m (middle) and 9996 m (long) of ascent	10	Phenomenological study
Holt, Lee [30]	To examine the individual experiences of participants in an ultramarathon	Canadian Death Race; 125 km of mountain trails; 5100 m of ascent; Cut-off time of 24 h	6	Phenomenological study
Simpson, Post [26]	To examine experience of ultramarathon runners	not race-specific	26	Phenomenological study
Hanold [14]	To understand how (1) the physical body of female ultramarathon runners becomes a desired body beyond the race and (2) this desired body produces multiple and complex subjectivities	not race-specific	8	Phenomenological study
